# Penton blooming, a conserved mechanism of genome delivery used by disparate microviruses

**DOI:** 10.1128/mbio.03713-24

**Published:** 2025-03-19

**Authors:** Pavol Bardy, Conor I. W. MacDonald, Paul C. Kirchberger, Huw T. Jenkins, Tibor Botka, Lewis Byrom, Nawshin T. B. Alim, Daouda A. K. Traore, Hannah C. Koenig, Tristan R. Nicholas, Maria Chechik, Samuel J. Hart, Johan P. Turkenburg, James N. Blaza, J. Thomas Beatty, Paul C. M. Fogg, Alfred A. Antson

**Affiliations:** 1 York Structural Biology Laboratory, Department of Chemistry, University of York, Heslington, United Kingdom; 2 York Biomedical Research Institute, University of York, York, United Kingdom; 3 Department of Microbiology & Molecular Genetics, Oklahoma State University, Tulsa, USA; 4 Department of Experimental Biology, Faculty of Science, Masaryk University 8748 , Brno, Czechia; 5 Department of Biology, University of York, York, United Kingdom; 6 Department of Microbiology and Immunology, University of British Columbia, Vancouver, Canada; 7 Materials and Structural Analysis, Thermo Fisher Scientific, Eindhoven, Netherlands; Duke University School of Medicine, North Carolina, USA

**Keywords:** electron microscopy, structural biology, *Microviridae*, virion structure, *Rhodobacter*

## Abstract

Microviruses are single-stranded DNA viruses infecting bacteria, characterized by T = 1 shells made of single jelly-roll capsid proteins. To understand how microviruses infect their host cells, we have isolated and studied an unusually large microvirus, Ebor. Ebor belongs to the proposed “Tainavirinae” subfamily of *Microviridae* and infects the model Alphaproteobacterium *Rhodobacter capsulatus*. Using cryogenic electron microscopy, we show that the enlarged capsid of Ebor is the result of an extended C-terminus of the major capsid protein. The extra packaging space accommodates genes encoding a lytic enzyme and putative methylase, both absent in microviruses with shorter genomes. The capsid is decorated with protrusions at its 3-fold axes, which we show to recognize lipopolysaccharides on the host surface. Cryogenic electron tomography shows that during infection, Ebor attaches to the host cell via five such protrusions. This attachment brings a single pentameric capsomer into close contact with the cell membrane, creating a special vertex through which the genome is ejected. Both subtomogram averaging and single particle analysis identified two intermediates of capsid opening, showing that the interacting penton opens from its center via the separation of individual capsomer subunits. Structural comparison with the model *Bullavirinae* phage phiX174 suggests that this genome delivery mechanism may be widely present across *Microviridae*.

**IMPORTANCE:**

Tailless *Microviridae* bacteriophages are major components of the global virosphere. Notably, microviruses are prominent members of the mammalian gut virome, and certain compositions have been linked to serious health disorders; however, a molecular understanding of how they initiate infection of their host remains poorly characterized. We demonstrate that trimeric protrusions located at the corners of a single microvirus capsomer mediate host cell attachment. This interaction triggers opening of the capsomer, driven by separation of subunits from its center, much like flower petals open during blooming. This extensive opening explains how the genome translocation apparatus, along with the genome itself, is able to exit the capsid. “Penton blooming” likely represents a conserved mechanism shared by diverse viruses possessing similar capsid architectures.

## INTRODUCTION

Viruses exhibit remarkable diversity in shape, symmetry, and size. For example, the genome sizes of giant viruses and microviruses differ by up to three orders of magnitude. In bacteriophages, the capsid encompassing the genome may be attached to a tail containing a host-recognition device called a baseplate, or it may exist without a tail, typically exposing spike protrusions at the external surface of the capsid that are used for host recognition. Although tailed phages have historically garnered the most attention, emerging metagenomic and environmental data are revealing a significant array of tailless phages (1, 2). Electron microscopy analysis of virion morphologies further suggests that these phages may dominate diverse environments (3). The *Microviridae* family, a major group within the realm of tailless phages, is abundant in aquatic habitats (4, 5) and terrestrial animal gut viromes, including those of humans (6). Variations in their abundance and diversity are linked to several disorders including inflammatory bowel disease, Crohn’s disease, and cognitive dysfunction (7, 8). The canonical *Microviridae* capsid protein shares the single jelly-roll fold with capsid proteins from a wide range of viruses, including eukaryotic RNA and DNA viruses, some of which are major pathogens. The capsids shelter a circular ssDNA genome ranging in size from 3.0 kb to 8.9 kb as suggested by metagenomic sequences (9, 10). The genome usually encodes 6–10 proteins, among which the major capsid protein VP1/F, DNA pilot protein VP2/H, internal scaffolding protein VP3/B, genome replication initiation protein VP4/A, and the ssDNA-binding protein VP8/J (11) stand out as most common.

Based on metagenomic data, the *Microviridae* family can be subdivided into over 20 groups, but only a handful of phages have been isolated, and far fewer have undergone structural analysis (1). The two virus subfamilies with officially assigned taxonomies (12) are the *Bullavirinae*, containing the model *Enterobacteria* phage phiX174, and the *Gokushovirinae*, with best-studied representatives including *Spiroplasma* virus SpV4 (13, 14), *Chlamydia* phage Chp2 (14), and coliphage EC6098 (15, 16). The key distinction between the subfamilies lies in their respective putative receptor-binding elements. *Bullavirinae* members interact with the host lipopolysaccharide (LPS) using a pentamer structure of the major spike protein G present at 5-fold symmetry axes of its virion (17). In contrast, *Gokushovirinae* phages lack the major spike protein and have instead evolved mushroom-shaped protrusions, formed by the major capsid protein VP1/F at the 3-fold symmetry axes (13, 16).

Our current understanding of the molecular mechanism of host infection by microviruses is based on cryo-EM structural analysis of *E. coli* phage phiX174 in complex with purified lipopolysaccharide (LPS) bilayers isolated from a member of a different bacterial genus, *Salmonella enterica* (17). According to this model, upon interaction with the LPS-core receptor, the extended pentameric structure of the major spike protein G somehow dissociates from the virion, whereas the virus remains attached to LPS. Following this, the hydrophobic loops of the major capsid protein become exposed and fuse with the LPS. The DNA pilot proteins located inside the virion rearrange and are thought to oligomerize to form a tube, which creates a channel for genome delivery into the host cytoplasm (18). Although the structural analysis of the phiX174-LPS complex pioneered our understanding of how microviruses penetrate bacterial cell walls (17), the proposed mechanism of infection remains to be probed by observing structural rearrangements of the virion during the infection of a native host cell.

Here, we describe the discovery and characterization of Ebor, an exceptionally large phage from the *Microviridae* family, along with a structural analysis of its genome delivery process. Ebor, a member of the proposed microvirus taxon “Tainavirinae” (19), infects the model aquatic Alphaproteobacterium *Rhodobacter capsulatus*. Its unusually large capsid size makes it an ideal system for the structural analysis of host cell infection by cryo-electron tomography. Subtomogram averaging of virus-infected *R. capsulatus* cells unveiled a mechanism whereby a capsid pentamer, nestled between *Gokushovirinae*-like trimeric protrusions interacting with the host, opens and peels back from the center. We refer to this mechanism as “blooming,” as the capsid subunits open outward from the center of the capsomer, like flower petals during blooming. These insights, integrated with previous structural data on phiX174 (17, 18), indicate that although different *Microviridae* subfamilies evolved to utilize distinct receptor-binding elements for host recognition, they are likely to employ a conserved penton blooming mechanism for capsid opening and genomic DNA delivery during infection. Although achieved by different means, the microvirus capsid “blooming” process is reminiscent of the “stargate” opening in giant viruses and may represent an evolutionarily convergent solution of capsid opening across a broad spectrum of viruses.

## RESULTS

### Discovery of a representative “Tainavirinae” member, Ebor

**Fig 1:**
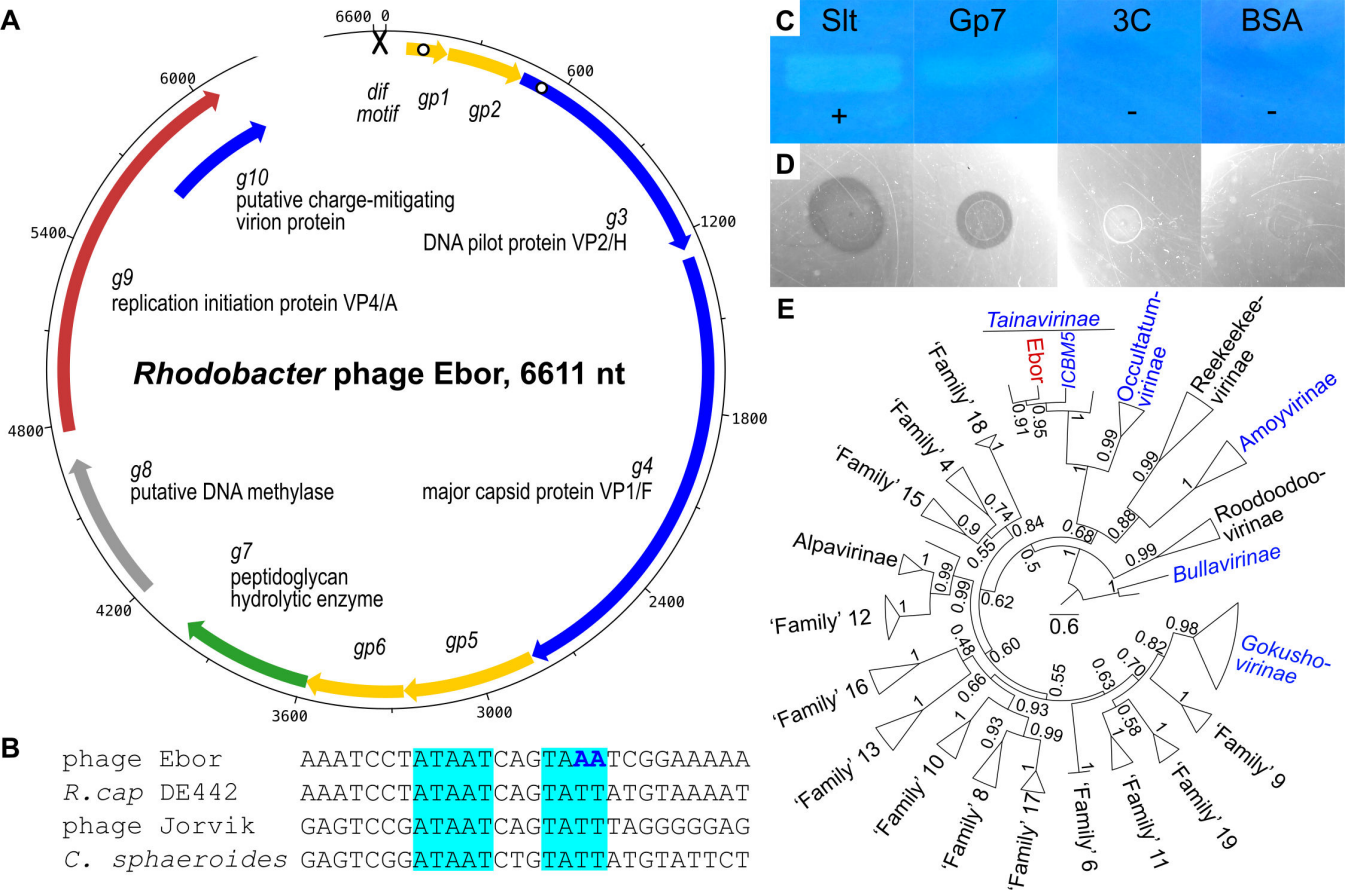
Overall characterization of the *Microviridae* phage Ebor. (**A**) Genomic map of phage Ebor depicting genes encoding structural proteins (blue), lysin (green), replication initiation protein (red), putative methylase (gray), and protein of unknown function (yellow). Cross designates the *dif* motif and white dots, locations of predicted transmembrane regions. (**B**) Alignment of putative XerCD/dif motif of Ebor with analogous sequences from *Rhodobacter capsulatus* DE442, *R. capsulatus* Jorvik (23) and experimentally verified motif from related species *Cereibacter sphaeroides* (25). The canonical *dif* AT repeats are highlighted in cyan, differing nucleotides in the imperfect motif of Ebor are in bold blue. (**C and D**) Zymogram (**C**) and water agar assay (**D**) of purified Gp7. Slt, a lysin from phage Jorvik (23); 3C, 3C protease; BSA, bovine serum albumin; plus and minus signs denote positive and negative controls, respectively. (**E**) Phylogeny of conserved major capsid protein calculated using sequences from up to three representatives from each major lineage, denoted by triangles. Taxa lineages with isolated members are in blue, Ebor is highlighted in red. Confidence values corresponding to 100 transfer bootstrap estimates are shown next to nodes. The scale bar denotes amino acid substitutions/site.

To investigate the genome delivery mechanism of microviruses, we established a tractable model system for the family. The *R. capsulatus* DE442 mutant strain, known for its elevated production of phage-like gene transfer agent (20), spontaneously produced phage particles that formed plaques on the parental *R. capsulatus* strain SB1003 (21). Isolation and sequencing of pure phage DNA using the Oxford Nanopore platform revealed a 6,611-base-long single-stranded DNA genome, to our knowledge, the largest isolated microvirus genome reported to date (Fig. 1A). The phage was named Ebor after the abbreviated Roman designation of the city of York, where this phage was isolated and described. The Ebor sequence was not identified in any of the complete genome sequences available in GenBank. However, sequence alignment with WGS genome entries of *Rhodobacter* strains, confirms that Ebor is transiently integrated as a prophage within the RuBisCO cbbII operon of *R. capsulatus* DE442, Y262, and R121 sister strains (22), using the same integration site as the dsDNA membrane-containing *Rhodobacter* phage Jorvik (23). Like in other ssDNA prophages, the integration site is flanked by XerCD-binding *dif* motifs (11, 24, 25; Fig. 1B). Ebor is absent from the parental SB1003 strain and its precursor, the wild-type strain B10 (26). Therefore, we conclude that Ebor had to be acquired from a laboratory environment during the construction of gene transfer agent overproducers in the 1970s (27) (see Fig. S1 at https://www.doi.org/10.5281/zenodo.14998181).

We identified 10 open-reading frames in the genome of Ebor (Fig. 1A; Table 1). These include the three most conserved genes of the *Microviridae* family: replication initiation protein VP4/A, DNA pilot protein VP2/H, and major capsid protein VP1/F (28). Mass spectrometry confirmed the presence of DNA pilot protein and major capsid protein in the sample of purified virionssee Data S1 at https://doi.org/10.5281/zenodo.14998181. The only other protein identified in significant amounts was the product of *g10*. This gene overlaps with the 3′-end of the gene encoding replication initiation factor VP4/A, and its product is highly positively charged with the theoretical pI of 11.3. It thus may have an analogous function to the conserved negative charge-mitigating ssDNA-binding protein VP8/J.

**TABLE 1:** Gene products encoded by phage Ebor^*a*^

Name	Function	Length (AA)	MW (kDa)	LC-MS/MS identification	Homolog (HHpred)^*b*^	Score	E-value	Residue range
Gp1	Membrane protein^*c*^	45	4.7	No	No	NA^*e*^	NA	NA
Gp2	Unknown	84	9.8	No	No	NA	NA	NA
Gp3	**DNA pilot protein VP2/H**	**265**	**28.2**	**Yes**	**P11336**	113.48	8.60E−15	2–61
Gp4	**Major capsid protein VP1/F**	**511**	**55.8**	**Yes**	**8DES_A**	596.67	3.30E−72	2–496
Gp5	Unknown	145	15.2	No	No	NA	NA	NA
Gp6	Unknown	107	12.4	No	No	NA	NA	NA
Gp7	Peptidoglycan hydrolytic enzyme	146	16.3	Yes^*d*^	6SSC_A	70.23	6.00E−11	9–144
Gp8	Putative DNA methylase	163	18.4	No	Q67472	78.81	2.30E−08	37–160
Gp9	**Replication initiation protein VP4/A**	**429**	**48.4**	**No**	**P03631**	266.31	7.00E−33	3–298
Gp10	Putative charge-mitigating virion protein	145	15.5	Yes	no	NA	NA	NA

^*a*^ The three signature products of the *Microviridae* family are highlighted in bold.

^*b*^ For each product the most relevant hit with an E-value below 0.1 is shown.

^*c*^ Predicted using TMHMM2.0.

^*d*^ Trace amount.

^*e*^ NA, not applicable.

In addition to structural proteins, Ebor encodes Gp7 and Gp8. These proteins possess similarities to peptidoglycan hydrolytic enzymes and a probable DNA methylase, respectively, based on the primary sequence alignment and subsequent comparison of profile hidden Markov models by HHpred (29) (Table 1). Purified Gp7 degrades crude *Rhodobacter* peptidoglycan in zymogram and water agar assays (Fig. 1C and D; see Fig. S2 at https://www.doi.org/10.5281/zenodo.14998181), and to our knowledge, Ebor represents the first experimentally verified ssDNA phage possessing a peptidoglycan hydrolytic enzyme. According to an analysis of nanopore sequencing data using the modkit tool, 11.53% of adenine calls belonged to 6mA, 1.58% of cytosine calls to 5mC, and 0.97% to 5hmC in any context. This proves that Ebor genomic DNA is methylated; however, we cannot assess if the methylation is performed by Gp8 or host methylases.

Taxonomic analysis with MOP-UP, a protein-sharing network algorithm using an up-to-date database of *Microviridae* genomes (1) (see Data S2 at https://www.doi.org/10.5281/zenodo.14998181), placed Ebor within the recently introduced subfamily “Tainavirinae” (19) and in a genus-level grouping with other *Rhodobacteraceae* prophages, which we confirmed by phylogenetic tree reconstruction (Fig. 1E). The genome composition of Ebor is typical of the “Tainavirinae” subfamily except for the putative methylase gene, which is only sporadically present in *Microviridae*. The conventional lytic gene is more conserved and shared among some members of “Occultatumvirinae” and “Tainavirinae,” which appear to be sister taxa (1). We note significant differences between “Tainavirinae” and isolates from other *Microviridae*. The only shared proteins with recognizable homology are the major capsid protein VP1/F, replication initiation protein VP4/A, and DNA pilot protein VP2/H. However, the amino acid identity for these shared proteins is below 30% (see Fig. S3 at https://www.doi.org/10.5281/zenodo.14998181).

### Extended protrusions at 3-fold symmetry axes of the capsid unfold during genome ejection

**Fig 2:**
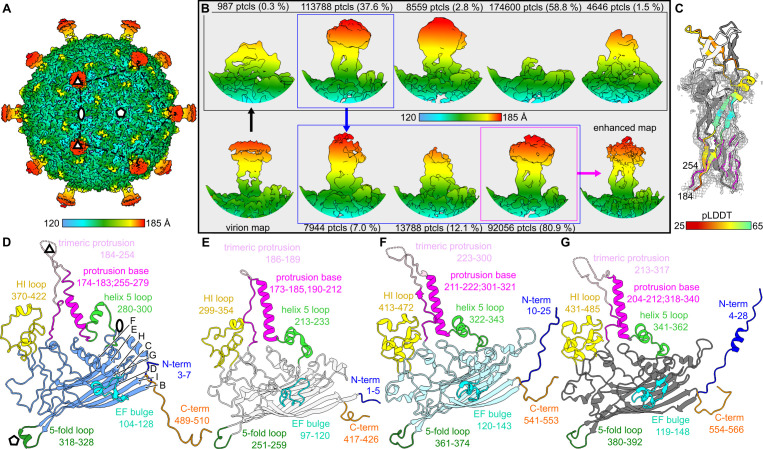
Structure of Ebor virion determined by single particle analysis. (**A**) Icosahedrally averaged map of the native capsid decorated with trimeric protrusions. The symmetry axes and position of one pentamer are highlighted. The color bar indicates the distance from the center of the capsid. (**B**) Symmetry expanded reextracted images of protrusions were subjected to two consequent rounds of 3D classification with the result of each round shown in black and blue rectangles, respectively. The best-resolved class (magenta rectangle) was enhanced by deepEMhancer (30). Maps are colored the same way as in panel A and shown in a similar density threshold. (**C**) AlphaFold2 model of the protrusion fitted in the enhanced map. One subunit is colored according to the prediction confidence measure pLDDT. The regions 183–192 and 250–254, which could be built in the experimental map are superimposed and shown in magenta. (**D–G**) Ribbon diagrams of the major capsid protein of Ebor (**D**); phiX174, PDB code 2BPA_B (**E**); SpV4, PDB code 9CGM_A (**F**); and EC6098, PDB code 8DES_A (**G**). β-Strands B-I are designated according to convention (31) and shown for Ebor. Regions of interest are highlighted in color, annotated with a description and delimiting residue numbers are shown.

We isolated two Ebor variants by successive propagation on strains B10 and SB1003, which exhibited different plaque morphologies. Nanopore sequencing of these variants showed a single nucleotide polymorphism leading to S120R amino acid replacement in the major capsid protein. Thus, we designated these variants S120 and R120, with the former displaying smaller plaques when plated on the strain SB1003 and the latter more turbid plaques when plated on the strain B10 (see Fig. S4 at https://www.doi.org/10.5281/zenodo.14998181). Ebor^R120^ virions were purified using CsCl gradient, vitrified and imaged using cryo-EM, yielding stable native particles. Single particle reconstruction produced an icosahedrally averaged map of Ebor at 3.2 Å resolution (Fig. 2A; see Table S1 at https://www.doi.org/10.5281/zenodo.14998181). The capsid shell has an external diameter of 28.0 nm and an internal diameter of 25.1 nm. The internal diameter is 3.2–4.6 nm larger compared with other determined capsid structures of *Microviridae* members, allowing Ebor to accommodate a larger genome (see Fig. S5 at https://www.doi.org/10.5281/zenodo.14998181).

A striking characteristic of the capsid is the presence of elongated protrusions situated at its 3-fold symmetry axes, reaching 9 nm from its surface (Fig. 2A through C). These extensions are formed by loops composed of residues 184–254 from three neighboring subunits of the major capsid protein (Fig. 2D). This is the same loop of the major capsid protein that forms trimeric protrusions in *Gokushovirinae* members (14, 16). As the density of the protrusions was resolved to a lower resolution, we employed a localized classification to identify a more resolved state of the protrusion (Fig. 2B). All identified conformations showed disordered density of 193–249 residues with various density levels and shapes. The atomic model of this region could not be confidently built in these maps, with the best class resolved at 5.1 Å resolution. A trimeric model of the loop generated using AlphaFold2 fitted reasonably well into maps except for the tip region that appears to be flexible (Fig. 2C; see Data S3 at https://www.doi.org/10.5281/zenodo.14998181). The model showed this tip possesses a positive electrostatic potential, in contrast to the negative potential of the capsid’s penton exterior (see Fig. S5 at https://www.doi.org/10.5281/zenodo.14998181).

**Fig 3:**
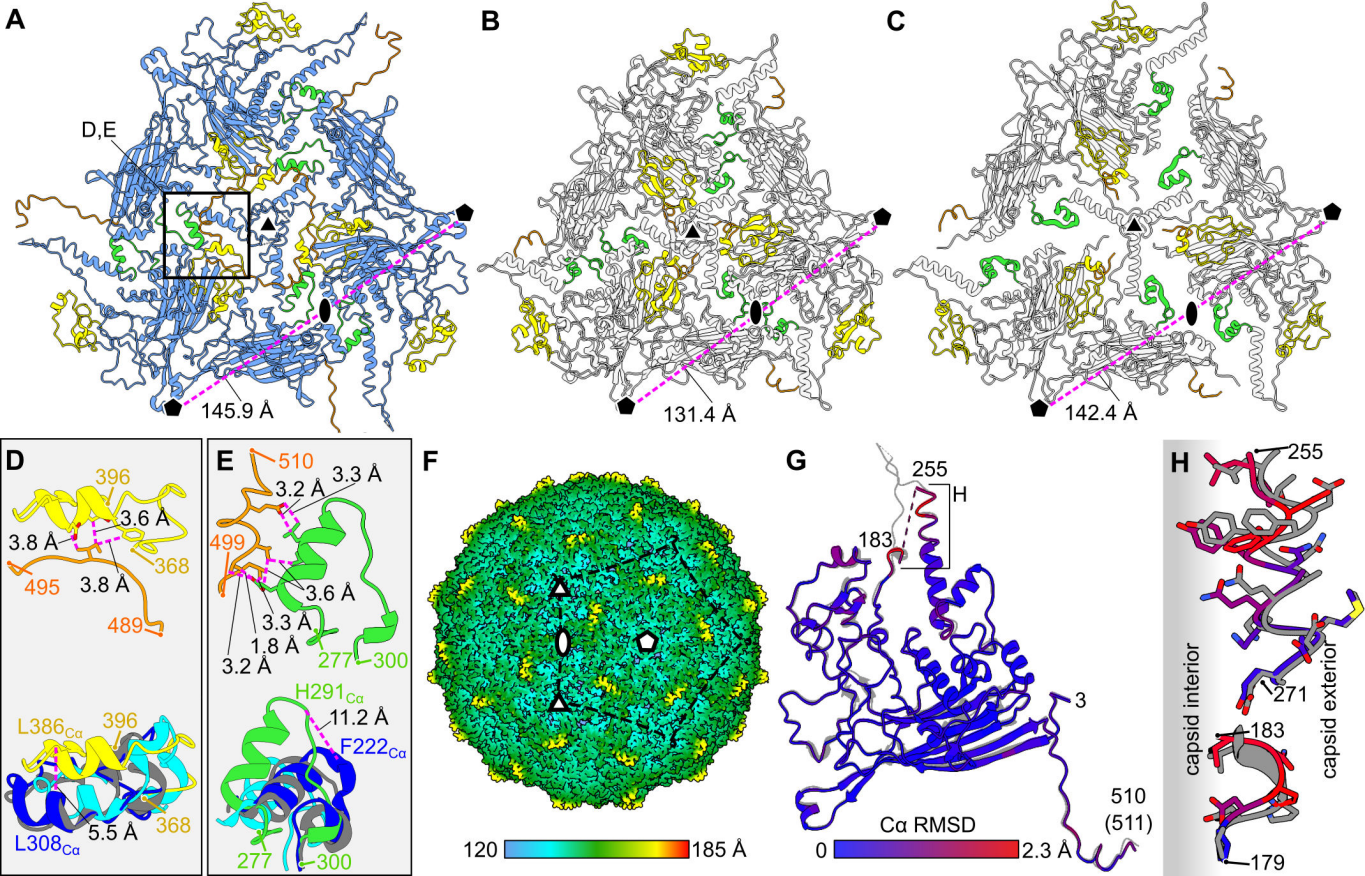
Structural basis for the enlarged capsid of Ebor and analysis of the empty particle. (**A–C**) Views of Ebor (**A**) and phiX174 (**B**) capsid proteins along the 3-fold axis of symmetry (triangle). The distance between the individual pentamer centers (penton) is shown. The capsid proteins of phiX174 superimposed to those of Ebor (**C**) highlight the void around 2-fold axes (oval), which is occupied by extended C-terminus, HI and helix 5 loops in Ebor virion (rectangle inset). (**D and E**) Interaction of HI loop (**D**) and helix 5 loop (**E**) with the C terminus (top images) causes their expanded conformation compared with the structures of phiX174 (blue), SpV4 (cyan), and EC6098 (gray) as shown by their superimposition (bottom images). The distances between the residues of loops interacting with the C-terminus (top) and Cα of corresponding residues of phiX174 and Ebor models (bottom) are shown. Delimiting residue numbers are shown for Ebor regions. (**F**) Icosahedrally averaged map of the empty capsid shows a missing density of protrusions. The color bar indicates the distance from the center of the capsid. (**G**) Superimposition of models built into the native (gray) and empty map (color). The color-coding is based on RMSD between corresponding Cα. (**H**) Close-up view of the protrusion base region where the largest deviations between the two models are present, shifting the residues to the capsid interior for the empty particle.

The capsid shell exhibits a triangulation number of T = 1, being made of 60 copies of the 511 residues-long major capsid protein (Fig. 2D). The core of the major capsid protein contains an eight-stranded antiparallel β-barrel (single jelly-roll fold), the fold shared among all *Microviridae* (Fig. 2E through G). The C-terminus of the major capsid protein is extended compared with other known structures of *Microviridae* members. Positioned beneath the loop connecting β-strands H and I of the adjacent subunit (as specified in phiX174 (31), referred to hereafter as the HI loop), it resides above the base of the protrusion and interacts with the helix 5 loop reported for phiX174 to bind sugars (32). The presence of extended C-terminus results in expanded conformations of the HI and helix 5 loops and leads to a greater curvature of the capsid compared with other studied *Microviridae* species (Fig. 3A through E; see Fig. S5 at https://www.doi.org/10.5281/zenodo.14998181).

Due to difficulties with preparing a more concentrated sample of Ebor^R120^ that could be used for an asymmetric reconstruction of the virion, we tested if Ebor^S120^ purified using the same protocol could result in a better cryoEM sample. However, this sample exhibited higher heterogeneity with DNA-like strings and some partially disassembled capsids present along with intact virions (see Fig. S4 at https://www.doi.org/10.5281/zenodo.14998181). Further analysis by two-dimensional (2D) and three-dimensional (3D) classification showed that ~10% of particles were missing one pentameric capsomer (see Fig. S5 at https://www.doi.org/10.5281/zenodo.14998181). A similar loss of a pentameric capsomer occurring *in vitro* was previously observed for enteroviruses and hypothesized to be linked to DNA ejection occurring through the 5-fold vertex (33). We subsequently employed sucrose gradient purification of Ebor ^S120^ and obtained a virus titer comparable with that obtained with CsCl gradient, as estimated by plaque assay contemporaneous with the cryo-EM grid preparation. However, examination of the grid showed that all the observed particles had ejected the genome (see Fig. S4 at https://www.doi.org/10.5281/zenodo.14998181). The ejection must have occurred during the blotting procedure, likely as an effect of the interaction with the air-water interface.

The icosahedrally averaged reconstruction of the empty particles led to a 3.3 Å resolution map, which notably lacked the trimeric protrusions (Fig. 3F). Apart from this major difference, only subtle changes were observed between the major capsid protein models, built into the maps of native R120 and empty S120 particles. The respective models exhibited an average RMSD_Cα_ of 0.55 Å with the maximal RMSD_Cα_ of 2.3 Å occurring at the base of the protrusion (Fig. 3G and H). Here, the residues move toward the capsid interior as they are not repelled by the pressurized genome, and we hypothesize this subtle change induces the unfolding of the metastable protrusion loop trimer. No density corresponding to two other structural proteins, the DNA pilot protein and Gp10, were observed in the averaged map of native nor empty particle. Since efforts to increase the count of stable particles in the micrographs were unsuccessful, we did not proceed further with an asymmetric reconstruction of the virion. We conclude Ebor^S120^ is less stable during grid preparation compared with Ebor ^R120^. Residue 120, located in the fusion loop, interacts with an adjacent subunit within the same capsomer. In the built models, S120 shows a higher solvation energy (0.03 kcal/M) compared with R120 (−0.43 kcal/M) at the interface of the interacting subunits, as analyzed by PDBePISA (34) and may explain the molecular basis of the observed instability.

### Ebor recognizes the host LPS as its receptor

**Fig 4:**
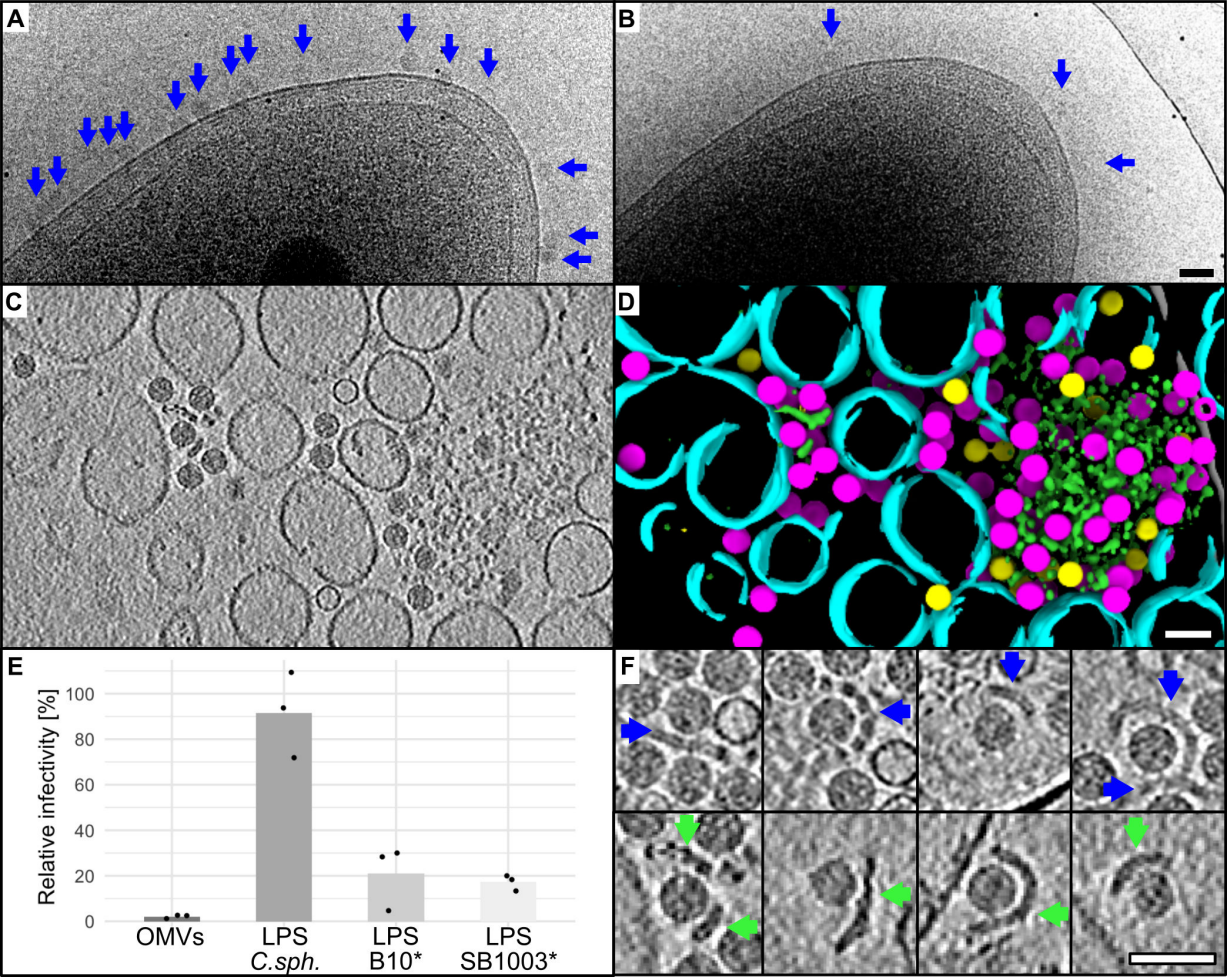
Characterization of host receptor. (**A and B**) Host cells, strains B10 (**A**), and SB1003Δ*gtaI* (**B**), imaged using Glacios TEM after 5 min incubation with the same stock of Ebor^R120^. The virions are depicted by blue arrows. Total exposure dose used was ~3 e^–^/Å^2^. (**C and D**) Central Z slice of a tomogram of Ebor virions mixed with purified OMVs (**C**) and its segmentation (**D**) highlighting native capsids (magenta), empty capsids (yellow), single-layered vesicles (cyan), and bilayered polymer (green). (**E**) Phage infectivity after incubation with different putative receptors. LPS, lipopolysaccharide; OMV, outer membrane vesicle; C.sph, *C. sphaeroides*; *, Δ*gtaI* strains. Individual measurements of biological replicates are plotted as points, and their averages are plotted as bars. The raw values are shown in Table S2 at https://www.doi.org/10.5281/zenodo.14998181. (**F**) Cryo-EM of four examples of phage particles attached to LPS bilayers isolated from B10Δ*gtaI* (blue arrows) and bilayered polymers present in OMV sample (green arrows). All scale bars are 50 nm.

We incubated susceptible host strains with the stock of Ebor^R120^ to determine the identity of the host receptor. Cryo-EM imaging revealed that phage particles were uniformly bound to the cell outer membrane (Fig. 4A and B), suggesting that the receptor is an abundant membrane component. To investigate further, we mixed Ebor^R120^ with purified outer membrane vesicles (OMVs) formed from disrupted B10 cells. Cryo-ET showed that the OMV sample consisted of single-layered vesicles 50–150 nm in diameter, larger single-layer membrane patches and bilayered polymers that tended to aggregate and attracted most of the phage particles (Fig. 4C and D; see Movie S1 at https://www.doi.org/10.5281/zenodo.14998181). LPS is an abundant outer membrane component, forms bilayers, can aggregate, and is a confirmed receptor for phiX174 (17, 35). We isolated LPS from *R. capsulatus* and tested whether it can competitively inhibit infection by Ebor. After 10 min, the Ebor titer dropped by 97.9% when incubated with OMVs and approximately 80% when incubated with LPS (Fig. 4E; see Table S2 at https://www.doi.org/10.5281/zenodo.14998181). Cryo-ET showed that the LPS sample also comprised bilayers that attracted phage particles, closely resembling the shape of the bilayered polymer present in the OMV sample (Fig. 4F). LPS extracted from two susceptible strains B10Δ*gtaI* and SB1003Δ*gtaI* inhibited the phage to a similar extent (Fig. 4E) and exhibited similar banding patterns on polyacrylamide gels (see Fig. S2 at https://www.doi.org/10.5281/zenodo.14998181). Intriguingly, SB1003 formed smaller plaques than B10 (see Fig. S4) and also bound considerably less phage per visible membrane area (Fig. 4A and B). Therefore, we predict that additional surface polymers are important for phage-host interactions or masking of phage receptors.

### Ebor binds to its host via five protrusions, inducing capsid penton opening

**Fig 5:**
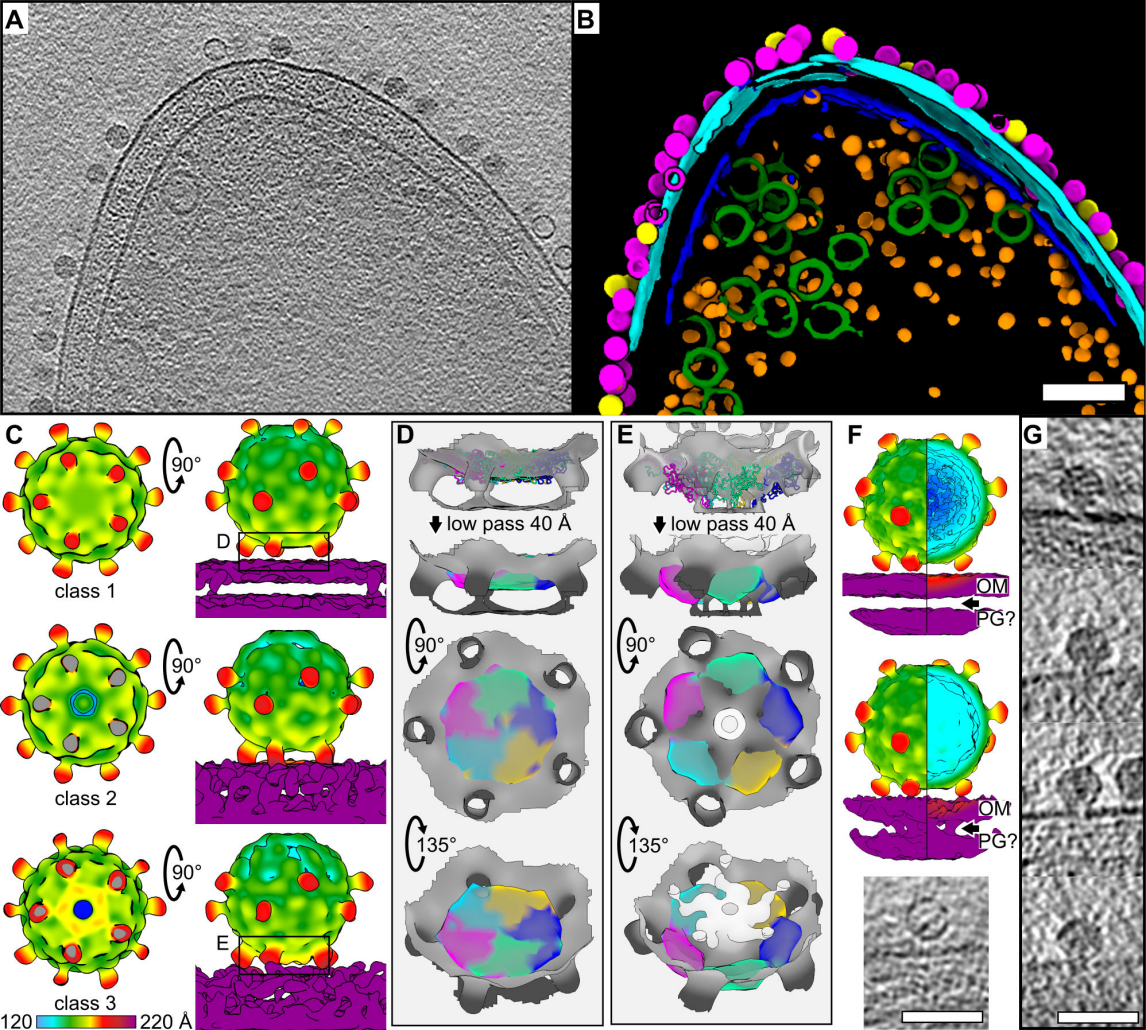
Cryo-ET analysis of Ebor-host cell interaction. (**A and B**) Central Z slice of a tomogram of *R. capsulatus* cell with attached virions (**A**) and segmentation of 127 nm-thick slab of the respective tomogram (**B**). Native (magenta) and empty (yellow) capsids, outer membrane (cyan), inner membrane (blue), intracytoplasmic membrane (green), and ribosomes (orange) are highlighted. The scale bar is 100 nm. (**C**) Two orthogonal views of C5 symmetry maps of the attached particles reconstructed by subtomogram averaging, classified into class 1 (top), 2 (middle), and 3 (bottom). Left, virions viewed from the membrane side, with the membrane cropped out and with a cross-section of virion-membrane connection sites (middle and bottom diagrams) coloured in gray. In the side views (right), the upper trimeric protrusions, present in all maps, are often invisible with the selected density threshold due to ice thickness gradient. The color bar indicates the distance from the center of the capsid. (**D, E**) Interacting penton densities of the class 1 (**D**) and class 3 (**E**) rigid body fitted with capsid protein models. A molecular map with a resolution of 40 Å was generated from the model to correspond to the achieved resolution. Three different views of the fitting are shown. (**F**) Maps derived from manually selected genome-containing (top) and empty (middle) particles. The arrows point to the space between the outer membrane (OM) and an associated layer that may be the peptidoglycan cell wall (PG). The color coding corresponds to panel C. An example Z-slice of an empty particle with channel density is visible in the OM and PG (bottom). The rest of the periplasm is too granular to assess visibility of any structures. The scale bar is 50 nm. (**G**) Z slices of representative particles from polished high-defocus tomograms showing the penton opening toward the membrane surface. The scale bar is 50 nm.

We reconstructed tomograms of Ebor^R120^ attached to native B10 cells (Fig. 5A and B; see Movie S2 at https://www.doi.org/10.5281/zenodo.14998181). Subtomogram averaging of the 1,593 particles attached to cells revealed that the phage binds to the outer membrane using five protrusions, causing the penton nestled between them to approach the host surface (see Fig. S6 at https://www.doi.org/10.5281/zenodo.14998181). This attachment pattern differs from that observed for Ebor^R120^ particles attached to OMVs, where only two protrusions were involved (see Fig. S7), likely due to differences in membrane curvature. By performing focused classification around the interacting region of the virus attached to the host cell, we identified three different states of the particles, with the interacting penton moving closer to the outer membrane and ultimately opening (Fig. 5C). To enhance the resolution of these intermediates and validate their existence through an alternative method, we collected a single particle data set on the same sample, targeting acquisition areas (36) on cells. This yielded 23,095 attached particles with correctly estimated membrane orientation (see Fig. S8 at https://www.doi.org/10.5281/zenodo.14998181). Within this subset, we identified three classes corresponding to those obtained by subtomogram averaging (see Fig. S9).

The first class represents Ebor attached to cells where no conformational change of the virion shell compared with the particle in solution occurs (Fig. 5C and D; see Movie S3 at https://www.doi.org/10.5281/zenodo.14998181). The density of protrusions is only weakly connected with the membrane. In the second class, the trimeric protrusions are in closer contact with the membrane, with a pore being formed along the 5-fold axis of the penton nestled between the protrusions (Fig. 5C). A comparable classification of a weak and strong connection of protrusions with the membrane density was observed for particles interacting with OMVs (see Fig. S7 at https://www.doi.org/10.5281/zenodo.14998181). The third class (class 3) showed the penton opens further, seemingly fusing with the membrane, and generating a pore extending from the capsid (Fig. 5C and E; see Fig. S6).

All classes show a pronounced density inside the virion corresponding to an unreleased genome. To classify another intermediate state, we manually inspected tomograms and separated particles that looked empty and reconstructed the average of 225 final particles. No difference in the virion shell compared with a reconstruction derived from full particles was observed (Fig. 5F), probably as subunits of the interacting penton return to the closed, energetically preferred conformation after the ejection is completed. Nevertheless, the adjacent cell membrane density of the map derived from empty particles contained a disk crossing the bilayer (Fig. 5F). A dense disk at the point of contact was also observed in the membrane interacting with capsid opening intermediates (see Fig. S9 at https://www.doi.org/10.5281/zenodo.14998181). This density may correspond to DNA pilot proteins embedded in the outer membrane. However, unlike for *E. coli* minicells infected by phiX174 (18), we could not identify a long pilot protein tube that would span the periplasmic space.

**Fig 6:**
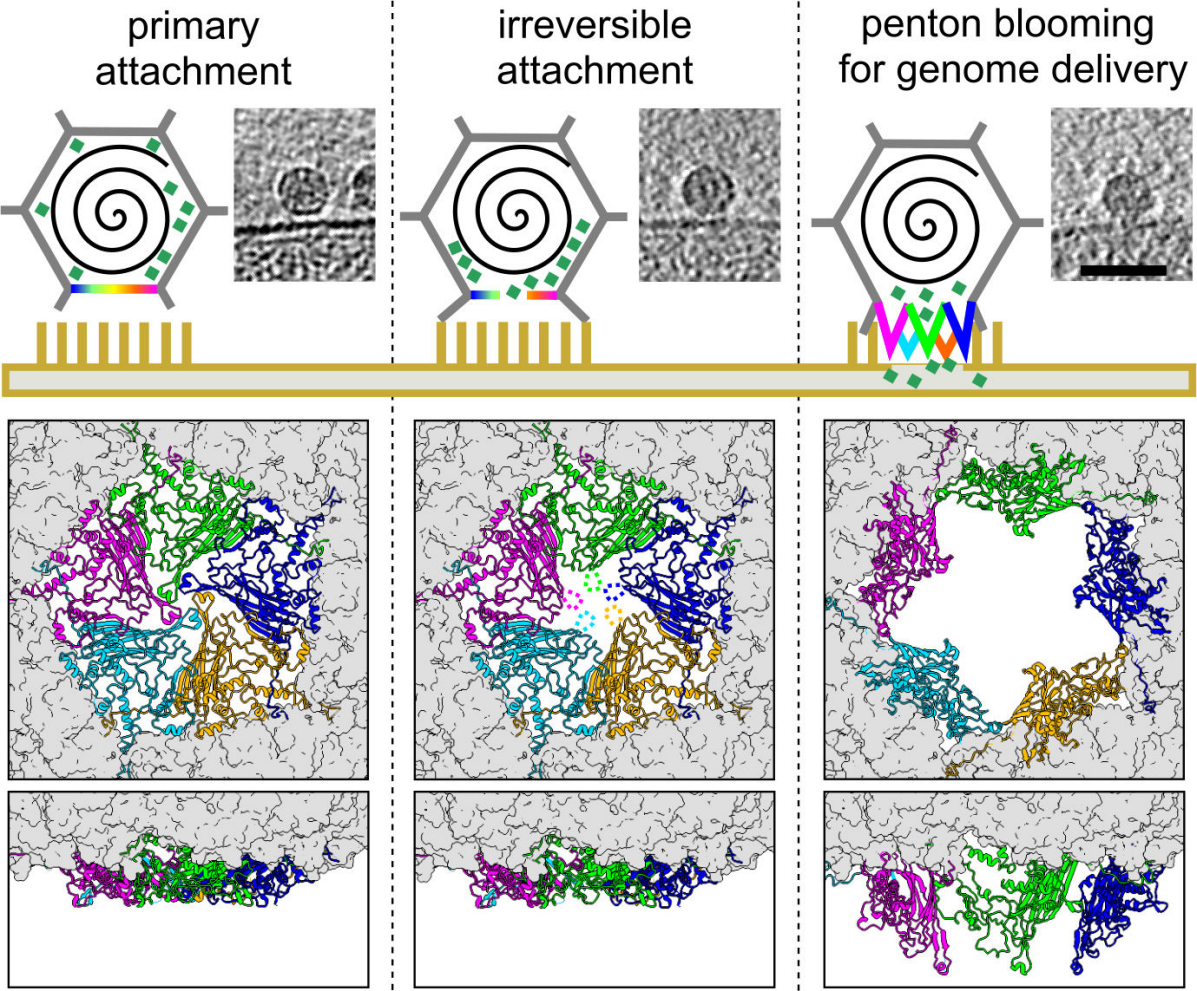
Mechanistic model of genome delivery. The three specific states, labeled at the top, correspond to classes 1–3 shown in Fig. 5C. Top row: Schematic illustration of three intermediate states of infection initiation, showing the virion (gray), interacting penton (rainbow), genome (black spiral), internal DNA pilot proteins (aquamarine squares), and membrane-imbedded lipopolysaccharides (yellow sticks). Corresponding examples of Z slices of particles from polished tomograms are shown to the right of each step. The scale bar is 50 nm. Middle row: capsid viewed toward the penton that opens up, with the central penton shown in colored ribbons and the rest of the capsid shown as molecular surface, gray. Bottom row: capsid that opens up viewed from the side, back half of the capsid is cropped for clarity.

Maps of class 3 generated by single particle analysis and subtomogram averaging differed slightly from each other. The map generated by the former method showed a smaller connection of the virion with the membrane, corresponding to the position of the EF bulge, with the central part of the interacting penton density being distorted (see Fig. S9 at https://www.doi.org/10.5281/zenodo.14998181). This map strikingly resembles that of phage phiX174 interacting with LPS (17) (see Fig. S9). In comparison, the map generated from subtomogram averaging showed a more pronounced bulging of the interacting penton into the membrane, suggesting adjustments in the position of entire subunits of the pentamer (Fig. 5E; see Movie S4 at https://www.doi.org/10.5281/zenodo.14998181). We note that a pronounced bulging of the interacting penton toward the membrane was also visible in single particles upon inspection of the polished tomograms (Fig. 5G; Fig. 6). An extra density is present around and above this bulging and likely correspond to rearranging DNA pilot proteins and/or genome (Fig. 5E; see Fig. S9). Fitting the capsid protein into the bulged density results in a virion model with a blooming of the interacting penton. In this model, the major capsid protein subunits forming the pentamer begin to separate from each other, gradually peeling away from the center of the pentamer (Fig. 6; see Movies S5 and S6 at https://www.doi.org/10.5281/zenodo.14998181).

## DISCUSSION

The identification of Ebor, the largest microvirus isolated to date, enabled accurate structural characterization of host infection by cryo-ET. Ebor is a member of a distinct subfamily within *Microviridae*, exhibiting substantial evolutionary divergence from the previously characterized *Bullavirinae* phage phiX174. This is exemplified by its considerably larger genome, 6.6 kb compared with phiX174’s 5.4 kb, although phiX174 seemingly utilize this space more efficiently, encoding 11 identified ORFs compared with 10 found in Ebor. To accommodate the enlarged genome, Ebor utilizes an expanded capsid with ~83% larger internal volume, built with the help of an extended C-terminus of the major capsid protein and several loops adopting a stretched conformation. The larger genome accommodates two genes not universally conserved within *Microviridae: g7*, bearing resemblance to peptidoglycan hydrolytic enzyme genes found in tailed phages, and *g8* that encodes a putative DNA methylase, likely contributing to phage resistance against host defense systems. Another notable feature of Ebor is the presence of the virion-associated protein Gp10, which is conserved within the ‘Tainavirinae’ subfamily. Its genome location and size are similar to the *Bullavirinae* scaffolding protein B (see Fig. S3 at https://www.doi.org/10.5281/zenodo.14998181). However, Gp10 is the only virion component identified that is highly positively charged, similar to VP8/J, which replaces the scaffolding protein when the ssDNA genome is loaded into the capsid of both *Bullavirinae* (37) and *Gokushovirinae* (15). The reason for encoding a larger internal protein Gp10 that contains 145 residues instead of a shorter VP8/J homolog (38–39 residues) is unknown, although evolutionary pressure to accommodate this may have contributed to the larger size of Ebor.

Based on structural findings, we postulate the following mechanism for phage Ebor genome delivery (Fig. 6). First, Ebor recognizes cell surface polysaccharides, including capsular polysaccharides and LPS; however, unlike the *R. capsulatus* gene transfer agent and related bacteriophages, the capsular polysaccharide is not an essential receptor (20). Second, Ebor irreversibly attaches to the cell via five trimeric protrusions of the major capsid protein that brings the phage closer to the outer membrane. This interaction instigates the formation of a pore in the phage capsid along the 5-fold symmetry axis of the interacting penton. Opening of the pore could be facilitated by a lack of intensive subunit-subunit interactions within the 48 Å diameter central region of the penton, that buries 1,788 Å^2^ of surface area. Finally, the interacting penton opens up, facilitating genome delivery. Given the rigidity of the β barrel forming the core of the major capsid protein subunit, it is reasonable to assume that the penton blooming occurs through the disruption of subunit-subunit interactions and their separation within the pentamer, whereas each subunit maintains interactions with the rest of the capsid. The energy required for this opening could potentially be generated by favorable interactions of the trimeric protrusions with the host’s membrane and the internal pressure within the capsid.

Notably, both maps of genome-releasing state belonging to class 3, derived from subtomogram averaging (Fig. 5C) and single particle analysis (see Fig. S9 at https://www.doi.org/10.5281/zenodo.14998181), showed that the virion’s penton fuses with the membrane. The map derived from subtomogram averaging aligns best with the blooming model, containing density for complete major capsid protein subunits. In the map derived from the single particle analysis, a vast portion of the penton density, including the highly stable core β barrel, appears weak and distorted. The inferior resolution is likely caused by the single particle acquisition of viruses attached to cells, being more prone to bias from preferential orientations. This bias arises predominantly because particles perpendicular to the incident electron beam are most amenable for analysis due to cell thickness. Similar bias was reported for the data set of phiX174 attached to LPS (17) with the derived map of the genome delivery intermediate also exhibiting a loss of the signal around the central penton, preventing precise localization of the respective major capsid protein subunits (see Fig. S9).

Despite specific differences between the capsid proteins of Ebor and phiX174 (Fig. 2D and E), maps of Ebor-cell and phiX174-LPS (17) interfaces are strikingly similar (Fig. 5C; see Fig. S9 at https://www.doi.org/10.5281/zenodo.14998181). This indicates that the general mechanism of capsid opening unveiled here is also applicable to the evolutionarily distant phiX174, and by extension likely to all members of the *Microviridae*. The initial interactions between the capsid and cell, priming the virus for penton blooming, are mediated by five trimeric protrusions, located at the corners of the interacting penton in the case of Ebor. Interestingly, initial attachment of phiX174 has been reported to occur via an extended major spike protein pentamer at the center of the interacting penton (17). However, the phiX174 capsid also has sugar-binding sites located close to its 3-fold axes, which become folded in the presence of calcium ions (32) and are essential for phiX174 host attachment (38). These sugar-binding sites may play a similar role as Ebor protrusions and would explain how the attachment of *Bullavirinae* phages is maintained before the penton opens up. This showcases a potential conserved role of 3-fold axes in the genome delivery process of *Microviridae*.

In phiX174, the internal DNA pilot proteins multimerize to form a DNA delivery tunnel (18). It was also shown that the genome-releasing intermediate contains a cavity with weaker density inside the capsid, hypothesized to be caused by the rearrangement of pilot proteins (17). We observed a weaker internal density right above the interacting pentamer in classes 2 and 3, suggesting ongoing rearrangement, albeit less prominent than in phiX174 where a diffused void cavity reaches the center of the capsid (see Fig. S9 at https://www.doi.org/10.5281/zenodo.14998181). We observed virion-associated disks spanning the outer membrane, likely corresponding to DNA pilot proteins. However, no distinctive features were visible extending deeper into the periplasmic space, possibly due to the flexibility of the tube and apparent high granularity of the space (Fig. 5F). Nevertheless, we cannot rule out that a different mechanism of genome translocation exists in Ebor and related phages, which may involve virus pilot proteins and/or host factors that facilitate genome translocation through the periplasm.

We conclude that genome delivery within the *Microviridae* family occurs via a general opening of capsid along a 5-fold symmetry axis, with LPS serving as a broadly recognized receptor and the protrusions at 3-fold axes serving as conserved receptor-binding elements. A mechanism reminiscent of penton blooming has been previously observed in giant dsDNA viruses at their special stargate vertex, where the capsid peels away from the 5-fold axis on a molecular scale involving many capsomers (39). Although the capsomer opening reported here occurs at a level of individual capsomer subunits, the macroscopic commonalities underscore the universal relevance of the penton blooming mechanism across different viral realms. This mechanism may also extend to other viruses built by single jelly-roll capsid proteins, such as the *Picornaviridae* family, which includes significant pathogens such as poliovirus. During infection, picornaviruses are endocytosed into the target cell and need to translocate their genome into the host cytoplasm. Penton blooming could explain how picornaviruses target their membrane-interacting peptides and genome toward the endosome membrane before the interacting capsomer is expelled (33, 40).

## MATERIALS AND METHODS

### Bacteria, media, and phages used in this study

The bacterial strains used in this study are summarized in Table S3 at https://www.doi.org/10.5281/zenodo.14998181. *R. capsulatus* strains were grown either in minimal medium RCV (41) or rich yeast peptone salts (YPS) medium (42), aerobically at 30°C. When working with the phage Ebor and strain SB1003, an additional 0.002 M CaCl_2_ was added to the liquid and soft agar media unless otherwise stated.

### Isolation of the phage Ebor and generation of phage variants

*R. capsulatus* DE442 was incubated in RCV medium at 30°C/200 rpm for 30 h, reaching a late log phase. The cells were pelleted at 4,000 × *g* for 6 min and 100 µL of the supernatant were added to 100 µL of stationary phase *R. capsulatus* SB1003. The mixture was overlayed onto YPS plates and incubated overnight at 30°C. A single plaque was picked using a sterile tip, resuspended in 100 µL of YPS medium and further propagated on a plate using the same propagation strain. After the second round of propagation, a spontaneous clear plaque appeared and was propagated on plates using strains SB1003 and B10, respectively. Two different strains were used for a separate propagation due to testing which one can provide a higher titer for subsequent purification experiments. After several rounds of propagation on strain B10, the phage stopped producing clear plaques on strain SB1003, likely as the result of the R120S mutation as identified by nanopore sequencing. This phage was designated as variant S120, whereas the phage propagated continuously on SB1003 was designated as variant R120.

### Production of Ebor in a high titer

Phage Ebor was washed from three confluently lysed plates using 5 mL of YPS, and this phage-containing medium was added to 25 mL of the propagation strain (SB1003 for variant R120, B10 for variant S120) grown to OD_600_ = 0.15. The mixture was incubated for 7 h at 26°C, 140 rpm. The supernatant was then added to 250 mL of the propagation strain grown to OD_600_ = 0.15 and incubated in the same manner. After this incubation, the titer of the lysate was in the range of 10^9-10^ PFU/mL. The lysate could be aliquoted to 0.5 mL and stored at −80°C for a year without an apparent loss of the titer.

### OMV extraction

The procedure was modified from Cian et al. (43). *R. capsulatus* was grown in 1 L of 1:1(vol:vol) YPS:RCV medium (44) to stationary phase and pelleted by centrifugation at 4°C, 6,900 × *g* for 15 min. The pellet was resuspended in 12.5 mL of 0.5 M sucrose in 10 mM Tris-HCl, pH = 7.5, supplemented with 114 µg/mL lysozyme and incubated on ice while stirring for 2 min; 12.5 mL of 1.5 mM ethylenediaminetetraacetic acid (EDTA) was added and stirred for 7 min. The mixture was centrifuged at 4°C, 11,400 × *g* for 10 min, and the pellet was resuspended in 25 mL of 0.2 M sucrose, 10 mM Tris-HCl, pH = 7.5, 2.2 mM MgCl_2_, 25 ng/µL DNase I with the addition of 1 µL of EDTA-free protease-inhibitor cocktail (MilliporeSigma). The mixture was homogenized with a Dounce homogenizer and lysed in French press (Aminco) at 4°C, 4 cycles at 20,000 psi. The lysate was centrifuged at 4°C, 6700 × *g* for 10 min, and the supernatant was further centrifuged at 4°C, 42,400 rpm for 90 min (Beckman 70Ti). The pellet was resuspended in 1 mL of 10% sucrose solution using a Dounce homogenizer and put on a step gradient of 2 mL of 73% sucrose, 4 mL of 53% sucrose, and 6 mL of 20% sucrose. The gradient was centrifuged at 4°C, 34,000 rpm for 45 h (Beckman SW-41), and the lowest band was collected, which was loaded on a second identical sucrose gradient for maximum removal of intracytoplasmic membrane (ICM) traces from the OMV fraction. The lowest band was collected once again and diluted 1:1 in 10 mM Tris, pH = 7.5 and pelleted at 4°C, 70,000 rpm for 60 min (Beckman TLA-100.3). The pellet was resuspended in 500 µL of 10 mM Tris, pH = 7.5 and kept on ice.

### LPS extraction

The procedure was based on Lam et al. (45). *R. capsulatus* B10Δ*gtaI* and SB1003Δ*gtaI* were grown in 1 L of 1:1 (vol/vol) YPS:RCV medium to stationary phase. The Δ*gtaI* strains that are unable to produce capsular polysaccharides (46) were used to ease the purification of LPS. Each culture was harvested by centrifugation at 4°C, 6,900 × *g* for 15 min. Pellets of approximately 1.5 g wet weight were resuspended in 10 mL of 100 mM NaCl and heated to 68°C. Ten milliliters of phenol prewarmed to 68°C were added, and the mixture was vortexed vigorously every 15 min for 1 h at 68°C. The mixture was cooled on ice for 10 min and centrifuged at 4°C, 12,000 × *g* for 15 min. The upper phase was transferred to a clean container. Ten milliliters of 100 mM NaCl were added to the lower phase, and the 68°C incubation and centrifugation steps were repeated. The pooled upper phases were dialyzed (in 3.5 kD cutoff tubing) against 3.5 L dH_2_O overnight and a second 3.5 L dH_2_O for 2 h. The sample was supplemented with MgCl_2_ (5 mM), DNase I (20 µg/ml), and RNase A (20 µg/mL) and incubated at 37°C for 1 h. Proteinase K was added (30 µg/mL), followed by incubation at 50°C for 2 h. The sample was lyophilized and resuspended in 4 mL of 20 mM NaOAc, pH 7.5, and centrifuged at 4°C, 200,000 × *g* overnight. The pellet was resuspended in 0.5 mL of 10 mM Tris-HCl (pH 7.5) and 0.1 mM MgCl_2_.

### Purification of Ebor using ultracentrifugation

From 500 mL of phage lysate containing 10^9-10^ PFU/mL, cells and cell debris were removed by centrifugation at 8,000 × *g* for 30 min. NaCl and PEG8000 were added to the lysate to a final concentration of 0.5 M and 8% wt/vol, respectively, dissolving at 4°C for 1 h. The sample was centrifuged at 4°C, 14,000 × *g* for 25 min, and the pellet was resuspended in 15 mL of E-buffer (20 mM Tris, pH 7.75; 100 mM NaCl; 5 mM CaCl_2_). For the caesium chloride ultracentrifugation, the sample after PEG precipitation was first spun at 10°C, 35,000 rpm for 3.5 h (Beckman Ty70.1Ti). The pellet was resuspended in 0.7 mL of E-buffer overnight and loaded onto handmade 1.3–1.4-1.45–1.5-1.7 g/mL caesium chloride density gradients. The sample was centrifuged at 12°C, 21,000 rpm for 5 h (Beckman SW41Ti). The band of the expected density was pooled using a syringe, and the buffer was exchanged to the E-buffer using 100 kDa Vivaspin columns (Cytiva). This method resulted in the production of purified Ebor^R120^ showing a titer in the range of 10^10-11^ PFU/mL that showed stable virions after vitrification and genome-releasing virions of Ebor^S120^ showing a titer 3 × 10^10^ PFU/mL (see Fig. S4 at at https://www.doi.org/10.5281/zenodo.14998181).

For the sucrose gradient centrifugation, the sample after PEG precipitation was loaded onto 15%–30% sucrose gradients prepared using Gradient Master (Biocomp) and spun at 15°C, 22,000 rpm for 1.5 h using Beckman SW28 rotor. The fractions containing the phage were pooled and spun at 10°C, 35,000 rpm for 3.5 h (Beckman Ty70.1Ti). The pellet was resuspended in 0.7 mL E-buffer overnight and loaded into 20%–40% sucrose gradients prepared using Gradient Master. The sample was centrifuged overnight at 10°C, 22,000 rpm (Beckman SW41Ti) with the phage sedimented at the bottom of the tube. The fraction was taken by cannula syringe, and the buffer was exchanged to the E-buffer using 100 kDa Vivaspin columns. This method resulted in the production of purified Ebor^S120^ reaching the titer of 5 × 10^10^ PFU/mL that upon vitrification contained only empty particles (see Fig. S4 at https://www.doi.org/10.5281/zenodo.14998181).

### Purification of Ebor using ion exchange chromatography

From 500 mL of phage lysate, cells, and cell debris were removed by centrifugation at 8,000 × *g* for 30 min. The supernatant was consecutively filtered through 0.8 and 0.4 µm filters. The filtered supernatant was mixed with the loading buffer (20 mM Tris, pH 7.75; 10 mM NaCl; 5 mM CaCl_2_) in the ratio 1:2 vol/vol and applied to a pre-equilibrated CIM-multus QA 8 mL column (BIAseparations). Particles of virus bound to the column were washed by applying 12 column volumes of the loading buffer followed by a linear gradient of elution buffer (20 mM Tris, pH 7.75; 1.8 M NaCl; 5 mM CaCl_2_) until the conductivity of the mixture reached 32 mS (~14% vol/vol). The elution of virus particles was induced by an increasing concentration of the elution buffer until the conductivity reached 40 mS (~16% vol/vol). The fractions containing the virus were identified using titering and pooled. The concentration of NaCl in the pooled sample was increased to 0.5 M and 8% (wt/vol) PEG8000 was added, dissolving at 4°C for 1 h. The sample was centrifuged at 4°C, 14 000 × *g* for 25 min, and the pellet was resuspended in the E-buffer. The sample was then aliquoted, frozen in liquid nitrogen and kept at −80°C. The sample showed a titer of 3 × 10^11^ PFU/mL, and when imaged, a mixture of native and empty particles in a similar ratio (see Fig. S4 at https://www.doi.org/10.5281/zenodo.14998181).

### Ebor inhibition assay

The concentration of LPS isolated from B10ΔgtaI and SB1003ΔgtaI and purchased LPS from *Cereibacter sphaeroides* (InvivoGen) was estimated based on the intensity of the LPS-corresponding band from the individual samples that were ran on a polyacrylamide gel stained using silver stain kit (Thermo Fisher Scientific) and alcaine blue (Millipore Sigma) (see Fig. S2 at https://www.doi.org/10.5281/zenodo.14998181). The samples were then diluted to equal concentrations of 0.5 mg/mL using ultrapure water, and *Rhodobacter* LPS was further buffer exchanged to ultrapure water using PES filter units with 5 kDa cutoff (VivaSpin). Here, the sample was diluted with water 1:1 and concentrated to the original volume with the process repeated four times to remove small molecule carbohydrates. In case of OMVs isolated from *R. capsulatus,* B10 and undiluted sample were used for the assay. For the phage sample, the ion exchange-purified phage was used in the assay. One microliter of the phage was added to 5 µL of the sample (OMVs, LPS, or ultrapure water) that was premixed with 50 mM Tris, pH = 7.5 to yield a final 10 mM concentration of Tris. The phage-sample mixture was incubated 10 min at room temperature and then diluted 100 times in YPS. Serial dilutions were made and dropped onto plates with soft agar containing 75 µL of overnight culture of SB1003. The plates were incubated overnight at 30°C, and the plaques were counted the next morning.

### Isolation, sequencing, and analysis of the phage genome

The genomic ssDNA was isolated from the ion exchange-purified sample of the phage using a Virus DNA kit (Macherey-Nagel). For sequencing on the Oxford Nanopore platform, libraries were prepared using the SQK-RBK114-24 Rapid Sequencing kit (Oxford Nanopore Technologies) according to the manufacturer’s instructions. The libraries were sequenced with a FLO-FLG114 flow cell in a MinION device controlled by MinKNOW software v.23.07.12, (Oxford Nanopore Technologies), which was also used for basecalling (Super-accuracy model for genome assembly, and Super-accuracy model for modified base detection for 5 mC, 5 hmC, and 6 mA in any contexts), demultiplexing and barcode trimming. Complete genome sequences were obtained using Flye v.2.9.1 (47) and Medaka consensus pipeline v1.7.2 (Oxford Nanopore). Methylation levels were analyzed by modkit v0.2.7 (Oxford Nanopore Technologies) using a 10% percentile threshold (A: 0.5996094, C: 0.60546875) for calls to pass. Only passed calls were included in the summary statistics. ORFs were identified using *ab initio* prediction of Prokka 1.14.5 (48) and GeneMarkS 4.28 (49) with the “phage” algorithm. The annotation and circular maps were done in Artemis (50). The predicted function of protein was based on primary sequence similarity using blastp (51), and the comparison of profile hidden Markov models using HHpred (29). The prediction of protein structures was performed using AlphaFold2 (52).

### Phylogenetic analysis

For initial taxonomic placement, protein sequences of Ebor were used as input to MOP-UP (53), which was run with minimum protein identity cutoffs of 30% and 50%. To construct a phylogenetic tree of the *Microviridae*, representative VP1 (major capsid protein) sequences for each microviral “family” as defined by Kirchberger et al. (1), and two additional sequences for phage Ebor and “Ascunsovirus oldenburgii” ICBM5 (19), were chosen. Proteins were aligned using ClustalOmega using the mBed algorithm (54) for initial and refinement iteration guide trees (cluster size 100) and 100 refinement interactions. After pruning sites with >50% gaps in the alignment, a phylogenetic tree was constructed from the resulting alignment in RAxML (55) using the PROT_GAMMA_GTR substitution model and 100 rapid bootstrapping replicates. Transfer bootstrap estimates were then assessed using the BOOSTER web interface (56).

### Proteomic analysis

The LC-MS/MS analysis was performed by The Centre of Excellence in Mass Spectrometry, University of York. Here, the samples of purified Ebor were run 1 cm into a 10% SDS PAGE gel and stained with SafeBLUE stain (NBS Biologicals). The stained gel segment was excised, destained, reduced with dithioerythritol, and alkylated with iodoacetamide. Protein was in-gel digested using Promega sequencing grade trypsin and incubated overnight at 37°C. Resulting peptides were analyzed by LC-MS/MS over a 1 h acquisition with elution from a 50 cm EN C18 PepMap column (Thermo Fisher Scientific) driven by an mClass Ultra Performance Liquid Chromatograph (Waters) onto an Orbitrap Fusion Tribrid mass spectrometer (Thermo Fisher Scientific) operated in data dependent acquisition mode. MS1 spectra were acquired at high resolution in the Orbitrap mass analyzer. MS2 spectra were acquired in TopSpeed mode in the linear ion trap after HCD fragmentation.

The LC-MS chromatograms were aligned using Progensis QI and a concatenated .mgf file searched against the *R. capsulatus* SB 1003 subset of the National Center for Biotechnology Information and the microvirus proteome with all translated ORFs as identified by ORFfinder (NCBI), appended with common contaminants. Peptide identifications were filtered to 1% false discovery rate using the Percolator algorithm before importing accepted identifications onto the chromatographic data in QI and matching identifications between runs. Data were further filtered to require a minimum of two unique peptides per protein. Quantification has been performed using a Top3 approach, taking the peak areas from the best three responding peptides as a proxy for each protein. The summed, integrated peak areas for the Top3 peptides are then converted to an estimation of relative molar percent within the sample by expressing each as a percentage of the sum total Top3 areas among all proteins.

### Production of the lytic enzyme and enzymatic assays

The *gp7* was cloned using HiFi assembly (New England Biolabs) of PCR-generated products with pETYSBLIC3c plasmid (57). The primers used for amplification were 5′ TCC AGG GAC CAG CAA TGA TCT ATC AAG GCA AAG ACC 3′ acting as a forward primer and 5′ TGA GGA GAA GGC GCG TCA AAG CCA GTC CAC CGA ATG 3′ acting as a reverse primer. The assembled plasmid was transformed into *E. coli* Stellar cells (Takara Bio), and individual colonies were verified for the presence of the insert by sequencing (Eurofins Genomics GATC). The plasmid was subsequently isolated from a verified colony and transformed to expression strain *E. coli* Rosetta (DE3) pLysS (Novagen). The expression was induced with 0.5 mM IPTG at 25℃. The pellet was resuspended in buffer Lys (20 mM Tris, pH = 7.5; 200 mM NaCl; 5 mM CaCl_2_; 20 mM Imidazole), sonicated using Sonoplus HD2070 (Bandelin) and purified using Super Ni-NTA resin (Generon). The lysate was incubated with the resin for 15 min and subsequently washed three times with buffer Lys. Then, modified buffer Lys with an increased Imidazole concentration to 250 mM was added to the resin, incubated for 5 min and the eluted sample was buffer exchanged using 10 kDa filter units to the original buffer Lys. The his-tag was cleaved overnight in the presence of 1 mM DTT and 20 mg of 3C protease. His-tag and the protease were removed from the protein samples by 20 min incubation with Super Ni-NTA resin (Generon). The sample was then loaded through a Superdex 75 10/300 column using the ӒKTA pure to separate the lysozyme from any excess protease, and the sample was then concentrated to 0.5 mg/mL using 10 kDa Vivaspin (Cytiva) filter units. Zymogram and plate lysis assays were adapted from Benesik et al., 2018 (58) and performed as described previously (23). In brief, the analyzed proteins were diluted in buffer Lys without imidazole to a concentration of 0.6 mg/mL. For the zymogram, protein samples were loaded into SDS-PAGE gels, containing 15% acrylamide and 200 µL of R. capsulatus SB1003 crude peptidoglycan. The gel ran at 150 V for 75 min, was washed three times for 15 min in fresh distilled water, placed in a fresh container with distilled water and incubated at 30°C/40 rpm for 3 h. The gel was then stained with a solution of 0.1% Methylene Blue, and 0.001 M KOH for 3 h and destained in distilled water. For the plate lysis assay, 500 µL of *R. capsulatus* SB1003 crude peptidoglycan was incorporated into 4 mL of 0.4% (wt/vol) dH_2_O agar and poured on a clean petri dish. Protein samples were then dropped onto the plate and incubated for 3–12 h at 30°C until clear zones of peptidoglycan degradation appeared.

### Cryo-EM and ET sample preparation and data acquisition

Four microliters of purified virus sample were applied onto glow-discharged Quantifoil R2/1 300 mesh copper grids and blotted using Vitrobot Mark IV (Thermo Fisher Scientific) for 2 s, blot force 0, wait time 8 s, and plunge frozen in liquid ethane. For the virus attached to cells, cells of the respective strain were grown until the stationary phase in RCV containing a quorum-sensing molecule O-acetylserine (4 mM). Then, 1.5 mL of the culture was spun at 1,000 × *g*/5 min, the supernatant spun at 14,000 × *g*/15 min, and the final pellet resuspended in 75 µL of RCV. Then, 1 µL of ion exchange-purified phage was added to 4 µL of cells and after 5 min of incubation mixed 1:2 (vol/vol) with 6 nm BSA-gold tracers (Aurion), which were transferred to RCV by Micro Bio-Spin six columns (Biorad). Four microliters of the mixture was applied onto glow-discharged Quantifoil R2/1 200 mesh copper grids. The grids were blotted from the back side by covering one pad of Vitrobot with parafilm. Vitrobot settings of blotting time 9 s, blot force 0, wait time 2 s, were used before plunge freezing in liquid ethane. A similar approach was used for freezing Ebor with LPS and OMVs, with the blotting time decreased to 5 s. All grids were screened using a Glacios TEM (Thermo Fisher Scientific) operated at 200 kV and equipped with Falcon 4 camera at the cryo-EM facility of the University of York. Automatic data acquisition was performed using microscopes at York, Eindhoven and eBIC (Harwell) as stated in Table S1 at https://www.doi.org/10.5281/zenodo.14998181. The raw data were acquired in EER format for Falcon 4(i) and TIFF format for K3, and motion corrected using RELION implementation of MotionCor2 (59, 60). The dose-weighting was performed using the same implementation in the case of single particle data and using the alignframes IMOD command (61) in the case of tilt series. This command was also used for generating stacks out of individual tilt images. For single particle data, the CTF estimation was performed using CTFFIND4 (62).

### Cryo-EM single particle analysis of the empty particle data set

The particles were picked using a template-based autopicking pipeline implemented in Relion 3.1 (60). For the template generation, 200 particles were manually picked, extracted and 2D classified. Resulting class averages were used as 2D templates for autopicking, identifying 3976 particles. The particles were extracted in an unbinned 256 px box and were subject to further 2D classification to discard bad particles. The subset of 2,880 particles was selected for further processing with applied icosahedral symmetry in the Relion I4 setting. The initial model was generated *de novo* using Relion 3.1. The 3D refinement was performed followed by 3D classification to discard bad particles. Then, a mask was generated using the volume segmentation tool in UCSF ChimeraX (63) and relion_mask_create command, which encompassed the density of the capsid shell. After another run of 3D refinement and classification, the final 1,984 particles were Bayesian-polished and CTF refined using Relion 3.1. The final map was improved using DeepEMhancer (30). FSCs of the final map are shown in Fig. S10 at https://www.doi.org/10.5281/zenodo.14998181.

### Cryo-EM single particle analysis of the native particle data set

The particles were picked using a template-based autopicking pipeline implemented in Relion 3.1 (60). For the template, the 3D map obtained from the empty particle data set was used. The autopicking identified 5271 particles, which were extracted in an unbinned 360 px box and subjected to further 2D classification to discard bad particles. The subset of 5,043 particles was selected for further processing with applied icosahedral symmetry in the Relion I4 setting. The map generated from the empty data set was low-pass filtered to 45 Å and used as an initial model. The 3D refinement was performed followed by 3D classification to discard bad particles. Then, a mask was generated using the volume segmentation tool in UCSF ChimeraX (63) and the relion_mask_create command, which encompassed the density of the capsid shell. After another run of 3D refinement and classification, the final 4935 particles were Bayesian-polished and CTF refined using Relion 3.1 (60, 64). The final map was improved using DeepEMhancer (30).

For localized reconstruction of the trimeric protrusion, particles from the final icosahedral refinement were symmetry expanded using relion_particle_symmetry_expand, and sub-particles were reextracted in 96 px box based on the original coordinates shifted by −124 px in x and 24 px in z directions. An initial model was generated by running relion_reconstruct on the extracted particles and used for 3D classification of the particles with skipped alignment and blush regularization (65). The class that showed the best-resolved protrusion density was subjected to another round of 3D classification with skipped alignment. The final density was enhanced using DeepEMhancer. FSCs of the final map are shown in Fig. S10 at https://www.doi.org/10.5281/zenodo.14998181.

### Cryo-EM single particle analysis of the genome-releasing particle data set

The particles were manually picked to begin training Topaz software for autopicking. The model was then used to auto-pick 4891 particles. The particles were extracted in an unbinned 360 px box, rescaled to 64 px, and subjected to 2D classification. Selected classes were refined in C5 symmetry against a *de novo* model generated using Relion 3.1 (60). One round of 3D classification was applied to further separate particles that lost the capsomer and autorefined (60). The FSC of the final C5 map is shown in

### Model building

The protein model was first predicted using AlphaFold2 (52) and fitted into the deepEMhanced map using UCSF ChimeraX 1.7.1 (63). The model was refined iteratively using real space fitting in Coot 0.9.8.5 (66) and real space refinement in Phenix 1.20.1 (67). Models were subsequently refined using NCS constraints and with the interacting partners in the virion to prevent inter-molecular atom clashes. During the iterative refinement process, the molecular geometry was monitored using the wwPDB validation server (https://validate.wwpdb.org) and MolProbity (68), suspicious outliers were fixed manually using Coot and further refined in Phenix. The refinement statistics are presented in Table S4 at https://www.doi.org/10.5281/zenodo.14998181. The interface analysis was performed using PDBePISA (34). Examples of model-to-map fit are shown in Fig. S10 at https://www.doi.org/10.5281/zenodo.14998181.

### Tomogram generation and subtomogram averaging

The tomogram generation, CTF estimation and subtomogram averaging were performed in EMAN2 (69). The particles were picked using reference-based picking and manually corrected to retain only particles attached to the sample and remove those present on carbon. The subtomogram averaging was performed as shown in Fig. S5 and S6 at https://www.doi.org/10.5281/zenodo.14998181. After the final reconstruction with icosahedral symmetry applied, the information about subtilt alignment was utilized to polish the tomograms using the e2spt_polishtomo.py command of EMAN2 (69). The final maps from classification were filtered using e2proc3d.py command according to their respective refinement map to minimize the low-resolution ice gradient effect on their density. Full and empty particles were manually separated in the .lst file of the final C5 refinement based on the inspection of tomograms in e2boxer. The subsequent reconstruction of particles in respective lists was performed using EMAN2 refinement with p3 iterations. FSCs of maps are shown in Fig. S11 at https://www.doi.org/10.5281/zenodo.14998181.

### Cryo-EM single particle analysis of the particles attached to cells

The particles were picked using Topaz (70) with the neural network trained on a set of particles manually picked from 25 random micrographs. The Fig. of merit for particle extraction was selected based on the manual inspection of 25 micrographs, the particles were extracted in an unbinned 256 px box and processed in RELION 5 (71) as shown in Fig. S8 at https://www.doi.org/10.5281/zenodo.14998181. The final maps were low pass filtered to their estimated maximum resolution using relion_image_handler command. The FSC of the final C5 map is shown in Fig. S11 at https://www.doi.org/10.5281/zenodo.14998181.

### Data visualization

The structural figures were created using UCSF ChimeraX 1.7.1 (63). The tomograms were visualized, and movies were made using IMOD 4.12 (61). The charts were plotted using the ggplot2 system of the package R (https://www.r-project.org/). The electrostatic potential was colored according to Adaptive Poisson-Boltzmann Solver (72). The segmentation of tomograms was done using EMAN2 (73).

## Data Availability

Ebor nucleotide sequence data are available in DDBJ/ENA/GenBank: PP754850.1. Raw LC-MS/MS data of purified Ebor are deposited at MassIVE: MSV000094685MSV000094685. Cryo-EM raw movies have been deposited to the Electron Microscopy Public Image Archive (EMPIAR) under codes 12148, 12153, 12156, 12168, 12170, and 12185 (see Table S1 at https://www.doi.org/10.5281/zenodo.14998181 for details); electron density maps have been deposited in the Electron Microscopy Data Bank (EMDB) under codes 50356–50356 (see Table S1 for details); and the fitted coordinates have been deposited in the Protein Data Bank (PDB) under codes 9FFH for the native and 9FFG for the empty particle. The authors declare that all other data supporting the findings of this study are available within the article and its supplemental material (see https://www.doi.org/10.5281/zenodo.14998181) or are available from the authors upon request.
